# Role of immune checkpoint inhibitors combined with chemotherapy in recurrent drug‐resistant gestational trophoblastic neoplasia: Four case reports and literature review

**DOI:** 10.1002/cnr2.2016

**Published:** 2024-03-01

**Authors:** Weiqing Liu, Yukai Zhu, Ya Wang, Rong Li, Dongling Zou, Rui Chen, Lu Yang, Yu Huang

**Affiliations:** ^1^ Department of Gynecologic Oncology Chongqing University Cancer Hospital Chongqing China; ^2^ Department of Pathology Chongqing University Cancer Hospital Chongqing China; ^3^ Department of Medical Iconography Chongqing University Cancer Hospital Chongqing China

**Keywords:** case report, checkpoint inhibitors, chemotherapy, gestational trophoblastic neoplasia

## Abstract

**Background:**

Multiple studies have confirmed that programmed cell death 1 (PD‐1) and programmed cell death ligand 1 (PD‐L1) is widely expressed in gestational trophoblastic neoplasia (GTN) tissues. Therefore, immune checkpoint inhibitors may be an option for the treatment of recurrent and drug‐resistant GTN.

**Case:**

Four patients with recurrent or drug‐resistant GTN who were treated with PD‐1/PD‐L1 checkpoint inhibitor agents combined with chemotherapy were reported. The mean age of recurrence was 45.8 years (35–56 years), including three cases of choriocarcinoma (CC) and one case of invasive mole (IM). International Federation of Gynecology and Obstetrics (FIGO) prognosis score: ≤6 (low risk) in one case, 7–12 (high risk) in one case, ≥13 (very high risk) in two cases. There were two cases of lung metastasis and one case of vulvar and inguinal lymph node metastasis. One of the four patients underwent total hysterectomy and one patient underwent resection of lung metastases. All the four patients received comprehensive treatment of immunotherapy combined with chemotherapy after relapse, among which one patient achieved complete response (CR), two patients achieved partial response (PR), and one patient developed progressive disease (PD). Three patients who achieved PR or CR were maintained by single agent immunotherapy after combination therapy, and there was no disease recurrence during follow‐up. One patient with PD also achieved CR after using salvage chemotherapy after recurrence, and there was no disease recurrence during follow‐up. During the treatment, four patients had different degrees of immune‐related adverse reactions, all of which were grade I‐II, and no severe adverse reactions were found.

**Conclusion:**

Immune checkpoint inhibitors combined with chemotherapy has an impressive therapeutic effect on recurrent or drug‐resistant GTN with mild adverse reactions, which can be used as a treatment option for such patients. However, due to the lack of large sample data support, the specific time and treatment course of its use, long‐term use of adverse reactions and whether it affects fertility function remain to be solved.

## 1 INTRODUCTION

Gestational trophoblastic neoplasia (GTN) is a group of gestational trophoblastic diseases that involve abnormal proliferation of trophoblastic tissues, including invasive mole (IM), choriocarcinoma (CC), placental site trophoblastic tumor (PSTT), and epithelioid trophoblastic tumor (ETT). Patients with low‐risk GTN [International Federation of Gynecology and Obstetrics (FIGO) risk score ≤6 points] have a survival rate of practically 100%, and the survival rate of high‐risk (≥7 points) patients is about 80–90%. However, a small percentage of patients with GTN remain resistant to chemotherapy, leading to recurrence, metastasis, and ultimate death. At present, there is a lack of effective treatment for them. In addition, considering that the vast majority of GTN patients are in the reproductive phase, cytotoxic drugs inevitably cause female gonadal damages, which not only reduces the patient's fertility however also triggers long‐lasting functional effects such as premature ovarian failure or amenorrhea. For this reason, reducing the patient's drug resistance and improving the chemotherapy regimen are effective to improve the prognosis of patients with advanced recurrence and to protect the reproductive function.

Since immune tolerance is a determinant of normal pregnancy, many studies have confirmed that programmed cell death 1 (PD‐1) and programmed cell death ligand 1 (PD‐L1) are strongly expressed in GTN tumor tissues.^1^, ^2^ Immune blockade of PD‐1/PD‐L1 pathway may have the potential to reverse trophoblast tolerance in GTN. Given that the first record of PD‐1 inhibitor pembrolizumab for the treatment of drug‐resistant GTN in 2017,^3^ several studies have actually revealed the remarkable anti‐tumor effectiveness of pembrolizumab or avelumab in patients with resistant and relapsed GTN, indicating that PD‐1 inhibitors alone or in combination with chemotherapeutic agents may be a good strategy for the treatment of relapsed GTN, although there is still a lack of large‐scale clinical evidence.^4^ In this paper, we reported the results of four patients with relapsed GTN who were treated with PD‐1/PD‐L1 checkpoint inhibitor combined with chemotherapy at a single center (Chongqing University Cancer Hospital), the last follow‐up time of them was in July 2023.

## 2 CASE PRESENTATION

All patients were diagnosed with GTN based on medical history, human chorionic gonadotropin (hCG) level and histopathology. Their demographic data are summarized in Table 1, and the details of chemotherapeutic regimens are given in Table 2. The patients were initially treated at other hospitals and were admitted to our hospital for relapse.

**TABLE 1:** Summary of patient baseline characteristics and prior therapies.

	Patient 1	Patient 2	Patient 3	Patient 4
Age	56	51	41	35
Obstetric status	G8P4	G5P2	G7P2	G3P0
Diagnosis	CC	IM	CC	CC
Antecedent pregnancy (years)	17	2	4	6
Sites of disease	Uterine, pulmonary	Uterine, pulmonary	Pulmonary, inguinal, vulval	Uterine
FIGO score (last diagnosis)	14	5	16	8
Treatment lines	Prior chemotherapy regimens (cycle)			
1	5‐FU + Act‐D (2)	5‐FU (3)	Methotrexate (3)	Methotrexate (6)
2	TP + TE + sintilimab (3)	EMA‐CO (5)	EMA‐CO (2)	EMA‐CO (4)
3	EMA‐CO + sintilimab (6)	FAEV (3)	EP‐EMA + sintilimab (6)	TP + camrelizumab (5)
4		EMA‐EP + sintilimab (5)	TP/TE + sintilimab (1)	5‐FU (1), FAEV (5)
Surgery	Hysterectomy	Thoracoscopic Segmentectomy		
Serum hCG before using the anti‐PD1 agent	5396	233.69	25450.55	2583
Adverse events (grade)	Malaise (1)	Aspartate aminotransferase increased (1)	Nausea (1) Vomiting (2)	Aspartate aminotransferase increased (1)

**TABLE 2:** Treatment regimens.

Regimen	Agent and dose
5‐FU+Act‐D	26 mg/kg fluorouracil D1‐6
	6 μg/kg actinomycin‐D D1‐6
TP+TE	
TP	135 mg/m^2^ paclitaxel D1
	60 mg/m^2^ cisplatin D1
TE	135 mg/m^2^ paclitaxel D15
	150 mg/m^2^ etoposide D15
EMA‐CO	
EMA	0.5 mg actinomycin‐D D1, 2
	100 mg/m^2^ etoposide D1, 2
	300 mg/m^2^ methotrexate D1
CO	1 mg/m^2^ vincristine D8
	600 mg/m^2^ cyclophosphamide D8
5‐FU (intravenous)	30 mg/kg fluorouracil D1‐8
FAEV	2 mg vincristine D1
	100 mg/m^2^ etoposide D1‐5
	0.2 mg/m^2^ actinomycin‐D D1‐5
	900 mg/m^2^ fluorouracil D1‐5
EMA‐EP	
EMA	0.5 mg actinomycin‐D D1
	100 mg/m^2^ etoposide D1
	300 mg/m^2^ methotrexate D1
EP	150 mg/m^2^ etoposide D8
	75 mg/m^2^ cisplatin D8
Methotrexate	1 mg/kg methotrexate D1,3,5,7
EP‐EMA	
EP	150 mg/m^2^ etoposide D1
	75 mg/m^2^ cisplatin D1
EMA	0.5 mg actinomycin‐D D8
	100 mg/m^2^ etoposide D8
	300 mg/m^2^ methotrexate D8
TP	135 mg/m^2^ paclitaxel D1
	60 mg/m^2^ cisplatin D1
5‐FU (uterine artery interventional)	1 g methotrexate D1

**FIGURE 1:**
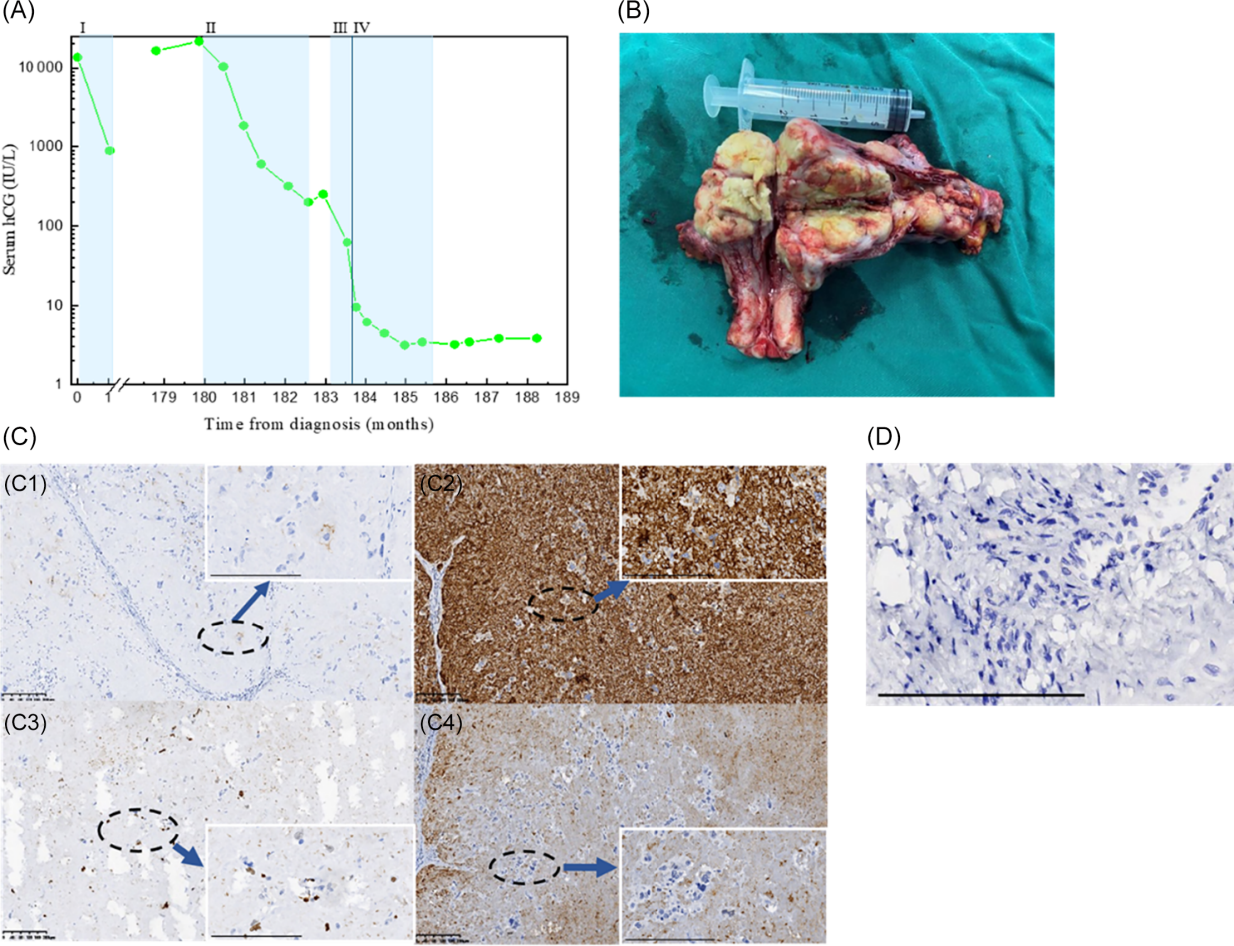
Clinical and pathologic characteristics of patient 1. (A) Serum hCG is plotted against time from treatment initiation. Shaded bands represent duration of chemotherapy and immunotherapy with I, 5‐FU + Act‐D; II, TP + TE + sintilimab; III, EMA‐CO + sintilimab; IV, laparoscopic hysterectomy and bilateral ovario‐salpingectomy. (B) Postoperative specimen after laparoscopic hysterectomy. (C) Immunohistochemical expression of PLAP (C1: ×100, ×400), HPL (C2: ×100, ×400), Ki‐67 (C3: ×100, ×400), and hCG (C4: ×100, ×400) in choriocarcinoma. Scale bar = 0.2 mm. (D) Immunohistochemical expression of PD‐L1 (×400) in choriocarcinoma. Scale bar = 0.2 mm.

Patient 1 was diagnosed with a hydatidiform mole (HM) at the age of 41 years and treated with curettage only. One year later, she underwent resection of uterine lesions due to abnormal vaginal bleeding. The pathological diagnosis was CC with bilateral lung metastasis, but the prognosis score was unknown. The patient discontinued treatment independently due to undisclosed reasons after receiving 2 cycles of actinomycin‐D (Act‐D) in combination with fluorouracil (5‐FU) in her local hospital. Fourteen years later, she was diagnosed with CC recurrence at our hospital because of vaginal bleeding and serum hCG elevation (16 383 IU/L). She initially received 3 cycles of paclitaxel, cisplatin/paclitaxel, and etoposide (TP/TE) regimen, and her hCG dropped rapidly from 16 383 to 201.38 IU/L after 2 cycles of treatment. However, since her hCG rose to 253.04 IU/L after the third cycle of treatment, we adjusted her regimen to etoposide, methotrexate, actinomycin‐D/cyclophosphamide, and vincristine (EMA‐CO) combined with 200 mg sintilimab once every 3 weeks [recombinant human immunoglobulin G (IgG4) PD‐1 monoclonal antibody developed in China (Innovent Biologics)]. After 2 cycles of combined regimen, her hCG dropped to 63.02 IU/L, and the hysterectomy was performed (Figure 1A,B). Immunohistochemistry (IHC) of uterine lesions showed positive expression of PLAP, HPL, and Ki‐67, and negative expression of hCG and PD‐L1 (Figure 1C,D). Tumor mutation burden (TMB, Beijing Genomics Institute) analysis showed TMB 3.94 Muts/Mb and microsatellite stable (MSS). Chest CT revealed that the residual lung lesions reduced from 26 × 19  to 25 × 18 mm and hCG was 9.63 IU/L, then followed by 1 cycle of combined regimen, her hCG decreased to normal (<5 IU/L). After 3 cycles of EMA‐CO combined with sintilimab for consolidation, chemotherapy was stopped, and sintilimab was given alone for 20 cycles. Her hCG remained normal during the following up, while the lung lesions persisted.

**FIGURE 2:**
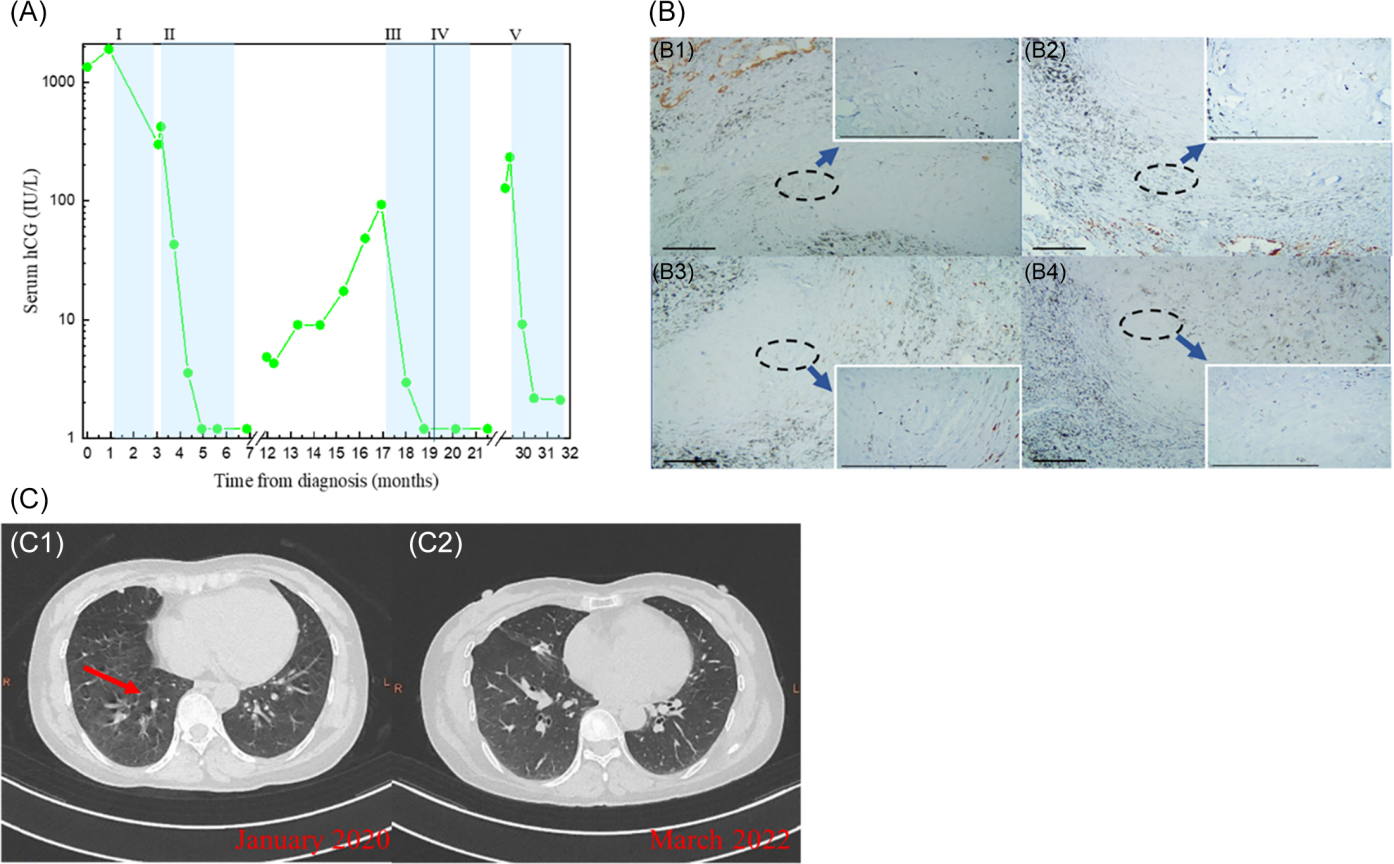
Clinical and pathologic characteristics of patient 2. (A) Serum hCG is plotted against time from treatment initiation. Shaded bands represent duration of chemotherapy and immunotherapy with I, 5‐FU; II, EMA‐CO; III, FAEV; IV, thoracic metastasectomy; V, EMA‐EP + sintilimab. (B) Immunohistochemical expression of CK‐pan (B1: ×100, ×400), TTF‐1 (B2: ×100, ×400), Ki‐67 (B3: ×100, ×400), and hCG (B4: ×100, ×400) in the lung metastases. Scale bar = 0.2 mm. (C) CT chest imaging shows resolution of lung metastasis (arrow). C1: January 2020; C2: March 2022.

Patient 2 was a 48‐year‐old woman who was diagnosed with IM (I: 7) in 2019 due to progressively elevated hCG (1899 IU/L) following curettage for HM. Specific medical information on her diagnosis and treatment at the local hospital were not available, except that she underwent 3 cycles of 5‐FU chemotherapy. After being referred to our hospital, she was diagnosed with IM (III: 5) with lung metastasis. Her hCG decreased from 299.56 IU/L to normal after 2 cycles of EMA‐CO chemotherapy, and the lung lesions disappeared. The patient additionally received another 3 cycles of EMA‐CO chemotherapy to consolidate the curative effect. However, her hCG increased to 93.08 IU/L and a 5 × 6 mm metastatic lesion appeared in the right lung after 5 months of chemotherapy. After receiving 1 cycle of the floxuridine, actinomycin‐D, etoposide, and vincristine (FAEV) regimen, the patient's hCG level returned to normal, and a thoracic metastasectomy was performed (Figure 2A). The pathological examination of the resected lesion showed inflammatory pseudotumor in the middle lobe of the right lung, and the local alveolar epithelium showed atypical hyperplasia. The pathologist opined that it was associated with gestational trophoblastic tumor metastasis (Figure 2B). After 2 cycles of FAEV regimen for consolidation, the patient experienced a progressive increase in hCG (233.69 IU/L) after 8 months of treatment, but no specific lesions were found in female reproductive organs or chest by positron emission tomography/computed tomography (PET/CT) or chest CT (Figure 2C). Meanwhile, we gave her 5 cycles EMA plus etoposide and cisplatin (EMA‐EP) chemotherapy combined with 200 mg sintilimab once every 3 weeks, her hCG returned to normal after 2 cycles of treatment, and she is continuing to receive sintilimab maintenance therapy until now.

**FIGURE 3:**
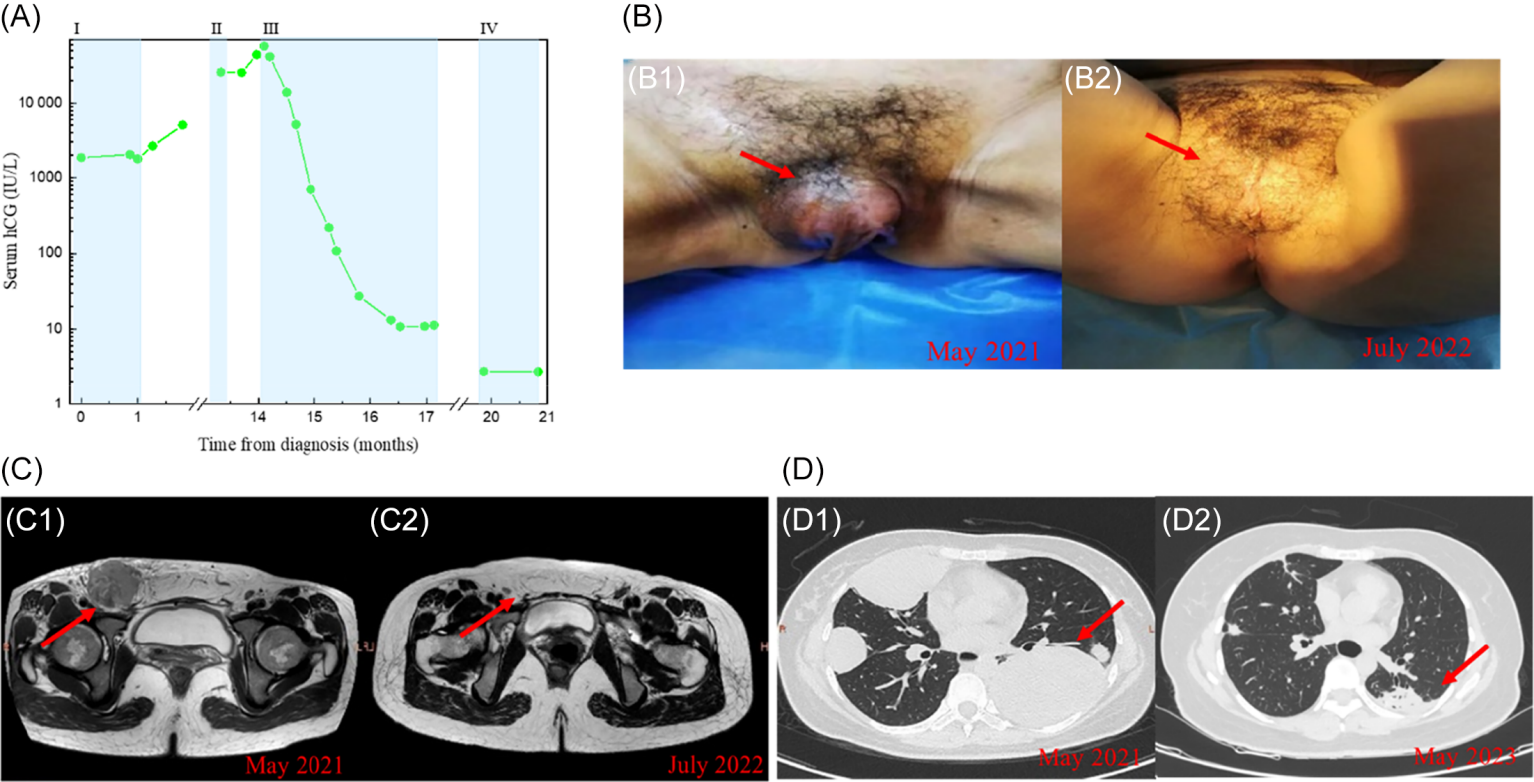
Clinical characteristics of patient 3. (A) Serum hCG is plotted against time from treatment initiation. Shaded bands represent duration of chemotherapy and immunotherapy with I, methotrexate; II, EMA‐CO; III, EP‐EMA + sintilimab; IV, TP/TE + sintilimab. (B) Gross appearance of the metastasis of vulva (arrow). B1: May 2021; B2: July 2022. (C) Pelvis enhanced scans show the reduction of metastases in vulva and right groin (arrow). C1: May 2021; C2: July 2022. (D) CT chest imaging shows resolution of a lung metastasis (arrow). D1: May 2021; D2: May 2023.

Patient 3 was a 37‐year‐old woman whose last pregnancy was an induced abortion. In April 2020 (more than a year after the induced abortion), she was found to have an abnormal hCG of 1859 IU/L, a 40 × 30 mm fusiform mass in the right groin, and a 15 × 15 mm mass in the vulva. The patient was diagnosed with CC and self‐discontinued treatment after 3 cycles of methotrexate. Eleven months later, the patient found that the vulva and groin masses increased significantly and her hCG increased (the exact value is unknown). After 2 cycles of EMA‐CO chemotherapy, her hCG dropped to 25450.55 IU/L and there were no significant changes in the masses. In our hospital, she was diagnosed with CC (IV: 16) with new bilateral lung metastases (the largest one was in the left lung, 50 × 50 mm) and received EP‐EMA regimen combined with 200 mg sintilimab for 6 cycles. Her hCG returned to normal after sixth chemotherapy. One cycle of TP/TE regimen combined with sintilimab was used for consolidation because of actinomycin‐D deficiency. Thereafter, the patient refused chemotherapy and only received sintilimab maintenance therapy (Figure 3A,B). Although her groin mass decreased from 58 × 36 mm to 5 × 4 mm, and the largest lung metastasis decreased from 50 × 50  to 36 × 17 mm (Figure 3C,D), the patient refused any surgery or biopsy. We therefore do not have any pathological information on her lesions. To date, she has received the treatment of sintilimab for 2 years, and her hCG remains normal.

**FIGURE 4:**
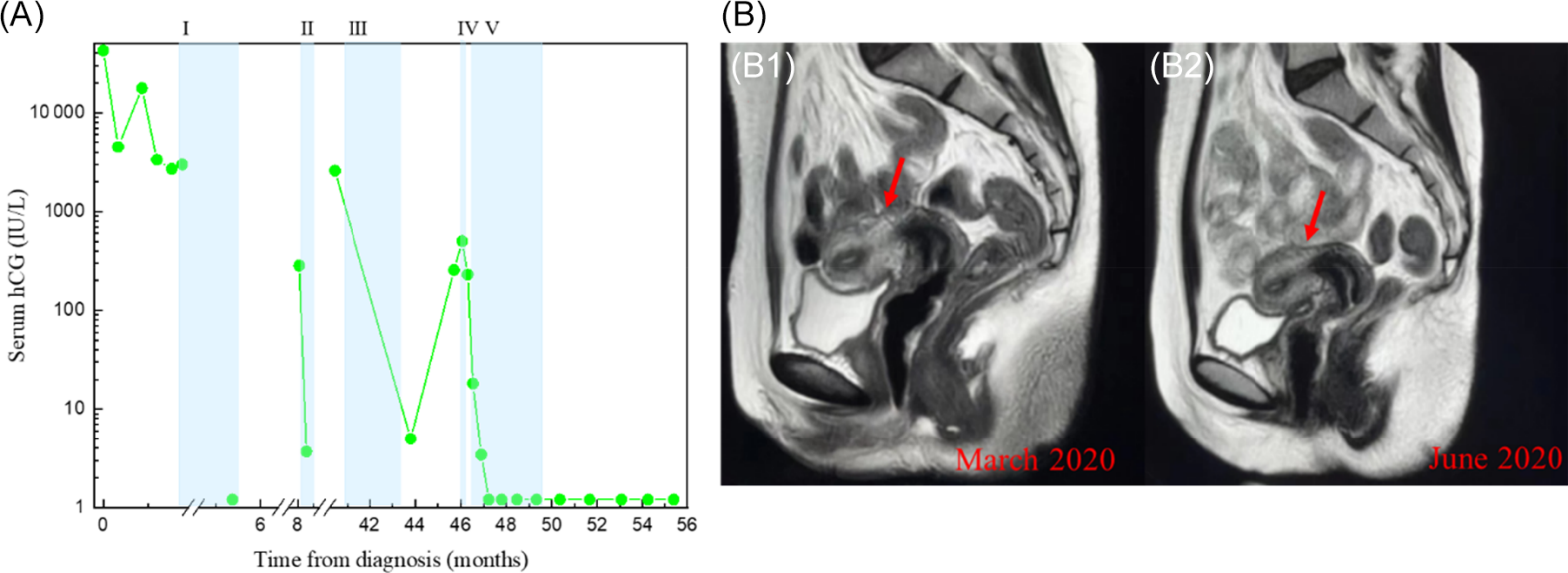
Clinical characteristics of patient 4. (A) Serum hCG is plotted against time from treatment initiation. Shaded bands represent duration of chemotherapy and immunotherapy with I, methotrexate; II, EMA‐CO; III, TP + camrelizumab; IV, Uterine artery interventional chemotherapy with 5‐FU; V, FAEV. (B) Pelvis enhanced scans show the reduction of myometrial nodules in the posterior wall of the uterus (arrow). B1: March 2020; B2: June 2020.

Patient 4 was diagnosed with IM when she was 29 years old. In the following four years, she received multiple different chemotherapeutic regimens including single methotrexate, EMA‐CO, TP combined camrelizumab [PD‐1 monoclonal antibody developed in China (Hengrui Medicine), 200 mg, once every 2 weeks] and FAEV (Figure 4A). Her first regimen was 6 cycles of intravenous methotrexate, with serum hCG rising to 283.92 IU/L after 3 months interval. The second chemotherapy regimen was 4 cycles of EMA‐CO and her hCG returned to normal. Two years later, she was found to have a 12 × 10 mm uterine lesion due to a menstrual disorder, and her hCG was elevated to 2993.63 IU/L. The patient was given 5 cycles of TP regimen combined with camrelizumab. After she finished the 5 cycles of chemotherapy, her hCG decreased to normal and uterine lesion disappeared. However, her hCG increased again to 256.8 IU/L, and the 11 × 10 mm uterine lesion was found by magnetic resonance imaging (MRI) after 2 months of treatment. At our hospital, 5‐FU was injected through the uterine artery, followed by 5 cycles of FAEV, and the patient remained in complete response (CR) for 37 months until now (Figure 4B). Her menstruation recovered to normal, but no pregnancy occurred.

All the patients tolerated chemotherapy combined with PD‐1 inhibitor or PD‐1 inhibitor alone well, and their adverse reactions (grades 1–4) are listed in Table 1.

## 3 DISCUSSION

Previous studies had shown that pembrolizumab or avelumab were effective in the treatment of recurrent or drug‐resistant GTN. In our study, four patients were treated with sintilimab or camrelizumab combined with chemotherapy achieved an impressive curative effect with mild adverse reactions. This provides a new treatment option for such patients.

The recurrence rate of GTN is extremely low. Previous literature reported that the majority of these patients (78%) recurred within 1 year after the completion of treatment,^5^ the greatest risk of recurrence is within 1 year in both high‐ and low‐risk patients.^6^ In the four cases we reported, the duration of recurrence varied: Case 1 and Case 4 had a long interval between recurrences (14/2 years), Case 3 was more susceptible to tumor persistence due to failure to adhere to regular treatment, while Case 2 had a short interval. The longer interval between recurrence may be related to the repeated and irregular chemotherapy treatment during the course of the disease, since GTN is a chemotherapy‐sensitive tumor. The situation of these four patients also reflects the necessity of long‐term monitoring of serum hCG, and follow‐up for 5 years should be considered.^7^

For resistant or relapsed GTN patients, salvage chemotherapy regimens are often selected based on their previous treatment. Generally, EMA/EP is an effective rescue regimen for EMA/CO‐resistant patients. According to Yang^8^ of Peking Union Medical College Hospital, FAEV was adopted as the main treatment regimen for patients with stage IV GTN, and 80% of patients achieved CR. In addition, the study of Feng^9^ showed that the CR rate of this regimen for drug‐resistant or relapsed GTN patients was 60.4%. On the basis of their study, we selected patients who had a good response to 5‐FU multi‐drug chemotherapy at the time of initial treatment, and administered FAEV regimen, which proved to be effective in Cases 2 and 4.

As mentioned above, PD‐1/PD‐L1 inhibitors are mostly used in high score or repeated drug‐resistant or recurrent high‐risk GTN cases. A recent phase II trial of camrelizumab plus apatinib in the treatment of high‐risk or recurrent GTN showed encouraging results with a median follow‐up of 18.5 months in 20 enrolled patients, an objective response rate of 55%, and no serious treatment‐related adverse events suggesting that camrelizumab is a new checkpoint inhibitor option for high‐risk or recurrent GTN.^10^ At the same time, another multi‐center retrospective analysis showed that compared with PD‐1 inhibitor monotherapy, PD‐1 inhibitor combined chemotherapy could significantly increase the CR rate of high‐risk or recurrent GTN from 54.3% to 87.1%, and patients who did not respond to PD‐1 inhibitor therapy could be effectively saved by salvage chemotherapy.^11^ In the report, case 1 to 3 continued maintenance therapy with immunosuppressive agents alone after combination therapy, and no recurrence has occurred to date. In Case 4, camrelizumab was discontinued immediately after 5 cycles of TP in combination, which may be related to rapid relapse for this patient. Recently, Gauci et al^12^ suggest that the recommended maintenance time for immunotherapy is at least 1 year if patient received the combination PD‐1 inhibitor and other chemotherapeutic agents.

Cytotoxic drugs play an immune‐enhancing role by upregulating the expression of PD‐L1 in tumor cells, inducing PD‐L1‐mediated tumor immunosuppressive phenotype, inhibiting negative immune signals, and changing the tumor immune microenvironment.^13^, ^14^ In turn, immune checkpoint inhibitors may also modulate the sensitivity of tumor cells to cytotoxic drugs. For example, it has been reported that partial response (PR) was achieved by albumin paclitaxel chemotherapy after the failure of PD‐1 inhibitor treatment with lung cancer patient.^15^ Anti‐PD‐1 monoclonal antibody not only enhanced the anti‐tumor effect of cisplatin on A2780/DDP cells but also effectively reversed the cisplatin resistance of platinum‐resistant ovarian cancer cells.^16^

Immune checkpoint inhibitors combined with chemotherapy were used in many tumors and proved safely. Xiang et al^11^ suggested that similar rates of grade 3–4 adverse reactions were observed in patients treated with anti‐PD‐1 monotherapy or combination therapy during the treatment of chemorefractory or relapsed GTN. Yin et al^17^ showed that neoadjuvant sintilimab plus platinum‐based chemotherapy was a safe approach in the treatment of locally advanced non‐small cell lung cancer (NSCLC) patients. Camrelizumab in combination with chemotherapy had also been used as a first‐line treatment for metastatic nasopharyngeal carcinoma, with 91% of patients achieving an objective response, but this was associated with a higher rate of grade 3 adverse events (87%).^18^ In our study, all the patients tolerated chemotherapy combined with PD‐1 inhibitor or PD‐1 inhibitor alone well, and no severe adverse reactions were found.

Before clinical application of immune checkpoint inhibitors, PD‐L1 expression, microsatellite instability‐high (MSI‐H), mismatch repair deficient (dMMR), TMB, tumor infiltrating lymphocytes (TIL), and other markers in tumor tissues are often used to predict the efficacy. Recent studies have found that plasma blood‐based TMB (bTMB) and circulating tumor DNA (ctDNA) are also associated with the efficacy of immunotherapy, and there is a correlation between these biomarkers. If the positive expression rate of PD‐L1 >1%, high expression of MSI‐H and TMB, or a large number of TILs, the tumor responds well to PD‐1/PD‐L1 inhibitor.^19^ In Case 1, although the tumor cell proportion score (TPS) of PD‐L1 tumor cells was 0, and the combined positive score (CPS) was <1, the patient achieved a good clinical response, which may be related to the effect of tumor tissue necrosis and inactivation after multiple lines of chemotherapy. However, there is no relevant data on the other three patients. Due to the particularity of GTN, it is very difficult to have access to fresh or chemical‐free tumor tissue. Given the favorable clinical outcome in this patient, it should be considered that the combination of immune checkpoint inhibitors may benefit the patients in in the presence of recurrent resistance, very high‐risk score, and long‐term recurrence even with negative biomarker testing or blinding.

The effects of immunotherapy on gonadal function and fertility are poorly understood. Polnaszek^20^ reported a patient diagnosed with stage I PSTT. She refused chemotherapy and surgery, and after 3 cycles of treatment with pembrolizumab, her serum hCG reverted to normal. Two weeks later, early pregnancy was detected, and pembrolizumab treatment was stopped, and the pregnancy continued until cesarean section at term. A normal baby girl was delivered successfully. In the TROPHIMMUN trial,^4^, ^21^ one patient received avelumab for 11 cycles, and became successfully pregnant afterwards and delivered a healthy baby. In our study, case 4 was the only woman who requested fertility, the effect of camrelizumab on her fertility was under evaluation.

Recurrent or advanced gynecological tumors are usually resistant to traditional cancer therapies. Immunotherapy has revolutionized the treatment strategy, and has been proven efficacious in various types of cancers. In addition, some other new treatments are being explored. For example, poly (ADP) ribose polymerase (PARP) inhibitors may be effective in newly diagnosed and recurrent ovarian cancer,^22^ the combination of human papilloma virus (HPV) therapeutic vaccines with radiotherapy, chemotherapy, or immune checkpoint inhibitors, may offer a new treatment option for locally advanced cervical cancer patients.^23^ These may also provide some new ideas for the treatment of recurrent or drug‐resistant GTN in the future.

## 4 CONCLUSION

Immune checkpoint inhibitors combined with chemotherapy can be a good option of recurrent or drug‐resistant GTN. Due to the low overall incidence of trophoblastic tumors and the lack of large sample clinical data, the timing of the addition of immune checkpoint inhibitors, the detection of predictive biomarkers, and the effect on perioperative and reproductive function remain unclear. Since the number of case reports in this paper is limited, the reversal of chemotherapy resistance in GTN patients need to be further explored in the future.

## AUTHOR CONTRIBUTIONS

**Weiqing Liu:** Conceptualization (equal); data curation (equal); investigation (equal); writing – original draft (equal). **Yukai Zhu:** Investigation (equal); software (equal); validation (equal). **Ya Wang:** Investigation (equal); software (equal); validation (equal); visualization (equal). **Rong Li:** Funding acquisition (equal); resources (equal); supervision (equal); writing – review and editing (equal). **Dongling Zou:** Funding acquisition (equal); resources (equal); supervision (equal); writing – review and editing (equal). **Rui Chen:** Data curation (equal); visualization (equal). **Lu Yang:** Investigation (equal); software (equal). **Yu Huang:** Conceptualization (equal); data curation (equal); funding acquisition (equal); resources (equal); writing – original draft (equal); writing – review and editing (equal).

## FUNDING INFORMATION

This study was funded by the 2022 policy consultation and Management Innovation Guidance Program of Chongqing Shapingba Science and Technology Bureau (No. jcd2022103).

## CONFLICT OF INTEREST STATEMENT

The authors declare that they have no conflict of interest.

## ETHICS STATEMENT

The patient provided written informed consent for the publication of this case report and accompanying images.

## Data Availability

The data that support the findings of this study are available on request from the corresponding author. The data are not publicly available due to privacy or ethical restrictions.
